# Making Worlds in a Waking Dream: Where Bion Intersects Friston on the Shaping and Breaking of Psychic Reality

**DOI:** 10.3389/fpsyg.2018.01674

**Published:** 2018-09-27

**Authors:** Matthew John Mellor

**Affiliations:** Kensington and Chelsea Psychotherapy Service, Central and North West London NHS Foundation Trust, London, United Kingdom

**Keywords:** Wilfred Bion, Karl Friston, Markov blanket, psychosis, alpha-function, contact barrier, free energy, dreaming

## Abstract

With the publication of Wilfred Bion’s text ‘Learning from Experience,’ psychoanalysis was afforded a new schema for understanding the processes and implications involved in an infant’s contact with their caregivers. As a result, our conception of some of the most fundamental phenomena of psychic life was significantly enriched. By proposing his theory of alpha-functioning, Bion mapped out how meaningful connexions to the internal and external worlds become established in the mind. In contrast, and through working clinically with psychotic patients, Bion revealed how these ties can catastrophically come undone. It is with these ideas, as well as their links to a corresponding set of neuroscientific constructs relating to the Markov blanket and principally developed by Karl Friston, that this paper is concerned. Through an investigation of the psychic functioning originally dubbed ‘dream-work-alpha,’ the paper’s first section focuses on how Bion conceived of the creation of a ‘contact-barrier’ that allows for the differentiation of consciousness from an unconscious mind. Casting the ramifications of this organisation in sharp relief, the psychotic disorganisation of the contact-barrier is then explored. The discussion subsequently broadens to incorporate contemporary theories from free energy neuroscience that bear significant and illuminating relations to the psychoanalytic ideas espoused by Bion over half a century ago. Finally, through posing a series of three questions with accompanying discussions, a superimposition of these theoretical schemas is attempted. These suggestions directly address, (1) whether there is an intimate connexion between the interoceptive contact-barrier and the exteroceptive Markov blanket, (2) whether a disobjectalising of the contact-barrier may be reflected as a tear in the functional fabric of the Markov blanket, and (3) what the clinical implications are of working at the level of the projected surface. Ultimately, the aim of the paper is to expose relevant points of contact within and between the varying conceptual frameworks; frameworks that ultimately derive from disciplines that are both concerned with examining the underlying mechanisms of the mind-brain.

## Introduction

In 1962, Wilfred Bion set out to deepen our conception of some of the most fundamental phenomena of psychic life by outlining the processes and implications involved in an infant’s contact with their caregivers. Elaborating on Melanie Klein’s notion of projective identification, Bion explored this contact in terms of the communication it facilitates (Segal, 2005). Going beyond parental contact and preverbal communication however, Bion’s theorising of ‘alpha-functioning’ offered psychoanalysis a new schema with which to understand how meaningful connexions to the external and internal worlds become established in the mind. By contrast, the insights he gained through working clinically with psychotic patients reveal how these ties can catastrophically come undone. It is with these ideas, as well as their links to a corresponding set of neuroscientific constructs relating to the Markov blanket and principally developed by Karl Friston, that this paper is concerned.

Through an investigation of the psychic functioning originally dubbed “dream-work-alpha” (López-Corvo, 2003, p. 91), the focus of the paper’s first section falls on how Bion conceived of the creation of a “contact-barrier” capable of “differentiating conscious from unconscious and maintaining the difference so established” (Bion, 1962a, p. 16). Casting the ramifications of this organisation in sharp relief, the psychotic disorganisation of the contact-barrier is then explored. The discussion subsequently broadens to incorporate contemporary theories from free energy neuroscience that bear significant and illuminating relations to the psychoanalytic ideas espoused by Bion over half a century ago. Finally, through posing a series of three questions with accompanying discussions, a superimposition of these theoretical schemas is attempted with a view to exposing relevant points of contact within and between the varying conceptual frameworks; frameworks that ultimately derive from disciplines that are concerned with examining the underlying mechanisms of “the same part of nature” (Solms, 2014).

## An Outline of Alpha-Functioning

At the very heart of this synthesis is Bion’s theory of alpha-functioning. For the infant faced with the task of developing a capacity for this, the prevailing external conditions play a pivotal role. Central to these conditions is the presence and temperament of the caregiver who, if able to effectively foster the infant’s mind after birth, engages in a way of being described by Bion (1962b, p. 309) as “maternal reverie.” In “good enough” (Winnicott, 1953, p. 94) conditions, this relationship allows the baby that possesses no “thought-thinking apparatus” to integrate their very first mental materials (Golse, 2003). Importantly for our concerns, Bion would come to classify these early emotional and sensory states as “beta-elements” that are liable for projection into the borrowed psyche of a “container” (Bion, 1962a, p. 6). Through the process of alpha-functioning, this containing figure is said to detoxify and transform beta-elements into “alpha-elements” that are capable of being assimilated by the infant (Golse, 2003). As Ferro (2011, p. 162) observes, the process equates to the conversion of proto-emotive chaos, into affectively meaningful representation.

Central to this exchange is the phenomenon of projective identification, the conventional definition of which is necessarily implicated provided the infant’s primitive anxieties can be contained and transformed by the caregiver in the way described. By contrast to being seen simply as a “fantasy *in* the infant’s mind” (Segal, 2005: my emphasis) where a psychological element is “displaced and relocated” (Laplanche and Pontalis, 1973, p. 349), the mechanism thus begins to resemble a search function that operates along the lines of a probe thrown into space (Pistiner De Cortiñas, 2011, p. 130). Where these projective probes encounter the kind of “transformational space” (Pistiner De Cortiñas, 2011, p. 130) that Bion had in mind, there exists the pregnant possibility of the baby’s “proto-emotive chaos” being metabolised or “digested” (Bion, 1962a, p. 7). With this perspective, an active transferral is seen to take place both within and between the container and the contained. What’s more, assuming this dyadic interaction can occur successfully and repeatedly, the infant ultimately stands to introject not only alpha-elements, but also their container’s *very alpha-function* (Pistiner De Cortiñas, 2011, p. 130). Crucially, it is this process that’s said to lead to the creation of a “contact-barrier”: an internal “membrane” which proliferates where alpha-elements cohere, owes both its manifestation and structural integrity to the developmental trajectory that alpha-functioning inculcates, and which ultimately “marks the point of contact and separation between conscious and unconscious elements” (Bion, 1962a, pp. 17–22).

For Bion (1962a, p. 17) the capacity to transform the sense impressions related to an emotional experience, into alpha-elements is described as continuous in both sleeping *and* waking states. Indeed, the original name for alpha-functioning – “dream-work-alpha” – goes some way towards overtly acknowledging this fact. For the purposes of this paper, an ability to ‘dream’ (in inverted commas) will be invoked with Bion’s meaning in mind; in other words that ‘dreaming’ reality works as a process of recording, assimilating and ‘digesting’ emotional experiences (Pistiner De Cortiñas, 2011, p. 136). Drawing on the theory that Freud had proposed in his seminal work on *The Interpretation of Dreams*, Bion suggests that the manifest content of a ‘dream’ should be considered as an enunciation that certain alpha elements are “constantly conjugated” (Pistiner De Cortiñas, 2011, p. 139). Understood as a context-sensitive adaption of Bion’s (1962b, p. 306) “constant conjunction,” a constant *conjugation* of alpha-elements reflects a clustered, connecting and combining agglomeration of the psychical products of dream-work-alpha. As such, one could say that we ‘dream’ the contact-barrier that creates “the distinction between the systems in the psychic apparatus” (Perelberg, 2005, p. 217); we *manifest* the “caesura”^1^ that moves us out from being solely under the sway of a wishfulfilling pleasure principle.

Turning briefly to address a further influence of Klein’s on this schema, establishing a contact-barrier capable of allowing both separation and communication between the psychic systems was for Bion (1962a, pp. 23/24), closely connected with “the change from paranoid schizoid to depressive position and vice versa.” As Bott Spillius et al. (2011, p. 78) note, it would take Bion’s insights to develop the Kleinian concept of the psychic positions to the extent of it becoming common to think of “a moment-to-moment fluctuation between paranoid-schizoid and depressive states of mind.” In positing the formula Ps↔D as reflecting the process of moving between psychic “disintegration and reintegration,” Bion showed that “oscillations” between these positions are not only normal, but also required for the very “development of thoughts” (Bion, 1963, p. 35). As Britton (1998, p. 69) explains in a manner that resonates with the aforementioned description of why alpha-function is necessary, “thinking arises to deal with thoughts; thoughts require containing, naming and integrating.” While ‘D’ involves producing a shape and containing it so as to imbue a meaning, the ‘Ps’ position must prevail for long enough for “the selected fact to emerge” (Britton, 1998, p. 69). Insofar as creative thinking resounds in the *coming into being* of integrating thoughts then, disintegration itself becomes an indispensable resource (Britton, 1998, p. 69).

Integrating these ideas into the present discussion and returning to *Learning from Experience*, we might suggest that the assimilation of a contact-barrier reflects a developmental “transition from a series of discrete particles or elements to a synthesis of these same elements” (Bion, 1962a, p. 24). In this sense, a transformation of beta-elements that had previously lacked “a capacity for linkage with each other” (Bion, 1962a, p. 22), into alpha-elements capable of constant conjugation, represents a binocular perspective on the transition from a world-view occupied by fragmented part-objects (Ps), to one where objects begin to be experienced ambivalently as separate and whole (D). It would fall to Segal (1957, p. 396) to highlight that this transition toward depressive integration has further consequences for an individual’s ability to *use* symbols. As she writes, only when separateness is accepted in the working-through of the depressive position does the symbol become “a representation of the object rather than being equated with the object,” the latter of which refers to the kind of symbolic equation synonymous with paranoid-schizoid functioning (Segal, 1981, p. 90). Recast in Bion’s language, an ability to conjugate alpha-elements by ‘dreaming’ corresponds to this capacity to transform incoherent masses of stimuli and sensory impressions into symbolised “ideograms” or “pictograms” that may be used to register present and future experiences (Pistiner De Cortiñas, 2011, p. 136).

In order to arrive at a full understanding of dream-work-alpha, this concept of the ideogram must feature as an essential component. In short, it testifies to the fact that alpha-elements serve as both an input *and* output of the process thus described. As Bion (1962a, pp. 6/7) observes, whether asleep or awake, “emotional experiences have […] to be worked upon by alpha-function before they can be used for dream thoughts […]. If the patient cannot transform his emotional experience into alpha-elements, he cannot dream.” In other words, an on-going capacity to transform latent dream thoughts into manifest dream content requires that assimilated nuclei of alpha-elements (ideograms) function as the unconscious imagos around which new conjugations of alpha-elements bind and cohere. While this process might be conceived of as latent dream thoughts connecting and cathecting at the contact-barrier, the overriding implication is that alpha-elements constitute both the raw material and yield of this “self-generating feedback system” (McGann, 1991, p. 15). Put yet more succinctly, alpha-functioning is therefore essentially autopoietic^2^.

## Alpha-Dysfunction and Psychotic Disorganisation

In circumstances where individuals exhibit a profound lack of dream thoughts, a principal factor to be considered would be whether the early environment was able to provide “good enough” conditions. Without a containing presence helping to provide the infant with auxiliary support in their attempts to digest beta-elements, the individual may ultimately have difficulty entering into the self-perpetuating system of dream-work-alpha outlined above. As Bion (1962a, p. 6) describes them, beta-elements “are not amenable for use in dream thoughts but are suited for use in projective identification.” In addition to being influential in producing acting-out, they are also used for the kind of thinking that depends on the “manipulation of what are felt to be things in themselves” (Bion, 1962a, p. 6). For the person incapable of transforming their experience symbolically, such a feeling of containing concrete things – rather than their images – can lead to the expectation of ideas behaving like sensory objects (De Masi, 2006, p. 20). As a result, these aspects of experience are liable to be split off and projected out of the mind in a manner that echoes the concreteness with which they’re felt within.

While these characteristics and phenomena are consistent with Klein’s account of the paranoid-schizoid position, a degree of elaboration is necessary if an adequately nuanced understanding is to be reached. Despite Klein’s (1935, p. 145) assessment that infantile paranoid-schizoid anxiety is “comparable to the psychoses of adults,” as Bion’s work exposes, ‘Ps’ functioning can also be regarded as necessary for the healthy development of thoughts. It must therefore be emphasised that the Bionian notion of a disintegrative ‘Ps’ position that can engender future growth is exclusively applicable in instances where ‘Ps’ exists *in dialectical tension with ‘D.’* In cases of profound early deprivation, a binary connexion between these positions may remain unestablished. Consequently, in the individual for whom the depressive position was never worked-through, there may be the pervasive lack of an ability to *use* disintegration resourcefully in the manner previously described; in other words, an incapacity to suspend attention with the kind of Keatsian “negative capability” that may creatively facilitate the formation of new realisations and new states of mind (Keats, 1952, p. 383).

Important considerations are thus raised around the psychic mechanisms at play in situations where severe early deprivation *is* experienced and where Klein’s comparison of paranoid schizoid anxiety to psychosis can be seen to resonate. As Winnicott hypothesises in *Fear of Breakdown*, when an infant endures extreme and traumatic conditions before they’ve developed sufficient perceptual apparatus to make sense of the overwhelming experience, psychotic defence organisations may be employed as a way to “short-circuit” the primitive agony (Ogden, 2014, p. 205). It’s here that, for Winnicott, the seeds of psychosis are sown. Moreover, by not experiencing the breakdown in the “mother-infant tie” when it occurs, the individual creates a psychological state in which they live in fear of a breakdown that has already happened, but which was not experienced (Ogden, 2014, p. 205). For clarification, the “mother-infant tie” that Ogden refers to here is assumed to be the integral factor in the process of generating alpha-functioning that was explored in the opening section of this paper. In addition, the psychotic disavowal that perpetuates an individual’s dissociation from the breakdown that was never experienced will, as Winnicott points out, be considered as a *communication* of the way the early environment failed (Winnicott, 1965, p. 128).

Should such a fundamental failure occur, the compulsion to avoid facing the pain of physical and emotional suffering can, Bion suggests, precipitate critically damaging consequences. As he puts it in his paper on the *Differentiation of the Psychotic from Non-psychotic Personalities*, the psychotic engages in the “minute fragmentation of the personality, particularly of the apparatus of awareness of reality” (Bion, 1957, p. 266). In other words, the very organs of emotional perception with which the experience would otherwise be registered may, in cases of extreme adversity, find themselves obliterated and eradicated. Moreover, such a wholesale attempt at neutralising pain can result in these fragments of the personality being expelled into external objects, where they become installed, often as a persecutory force (Bion, 1957, pp. 266–267). In Bion’s (1957, pp. 268–270) terminology, such patients may consequently feel themselves “to be surrounded by bizarre objects” that carry a disturbingly “menacing presence.”

By way of a clinical example of the bizarreness inherent in this psychic self-destruction, Bion, in his work *Cogitations*, recounts a patient who, “when unable to find the selected fact,” externalises the terrifying experience through the enunciation “blood everywhere” (Pistiner De Cortiñas, 2011, p. 143). Bion’s interpretive intervention in this instance was to convey that the patient had attacked their faculty for common sense which they thus saw spread everywhere as blood (Pistiner De Cortiñas, 2011, p. 143). What he was able to achieve with this insight and interpretation was the stemming of the tide by binding the spread fragments and formalising them into a scene (Pistiner De Cortiñas, 2011, p. 143). Cast in the language already prescribed, Bion lends his faculties and ‘dreams’ the “murder of common sense” (Pistiner De Cortiñas, 2011, p. 144) on behalf of his patient, thereby expressing his alpha-functioning and endowing the patient’s experience with significance and meaning (Bell, 2011, p. 94).

When working with patients of a psychotic disposition, Bion emphasises the importance that the analyst is able to provide such auxiliary support by lending their faculties and ‘dreaming’ the session on behalf of the patient. In this respect – and contrary to the view held by both Freud and Immanual Kant for whom “the madman” was regarded as a “waking dreamer” (Stevens and Price, 1996, p. 229) – Bion saw the madman as *requiring* a waking dreamer in order to ‘dream’ the thoughts he can’t. Furthermore, it is precisely this ‘dreaming’ (explored already as the capacity to consolidate alpha-elements), that provides the psychotic patient with invaluable containing tools for mental growth (Pistiner De Cortiñas, 2011, p. 140). In favourable circumstances, thoughts that previously lacked “a thinker” (De Masi, 2006, p. 51) may, within the carefully contained analytic situation, come to be circumscribed, symbolised and returned by the analyst to the agency from which they came.

Broadening the scope of the analytic technique under discussion and turning to a further Kleinian innovation, the process of playing, like ‘dreaming,’ can similarly be seen to produce constant conjugations of alpha-elements and facilitate the discovery of the selected fact (Pistiner De Cortiñas, 2011, p. 147). As Pistiner De Cortiñas (2011, p. 147) explains, playing within a clinical context can be used to harness feelings of guilt and criticism by encouraging patients to assign characters to the feelings previously experienced as “things in themselves.” Through playful transformation, patients are thus presented with the possibility of opening imagined dialogues with their internal worlds. As will be shown, these principles have been demonstrated to have profoundly positive impacts in terms of helping individuals suffering with psychotic symptoms. Avatar therapy, a form of mental health treatment developed at University College London and Kings College London, constitutes a psychotherapeutic method that arguably both embraces and enhances precisely this approach. Moreover, having been explicitly designed for individuals experiencing auditory or visual hallucinations, both of which are “Scheiderian first-rank symptoms” (Tandon et al., 2013, p. 2) of schizophrenia, the therapy is particularly relevant to the concerns of this paper.

Originally invented by Julian Leff in 2008, avatar therapy entails patients working with a therapist to create virtual representations of their internal persecutors (Craig et al., 2018, p. 31). These avatars are constructed using specialised modelling software in order to bear as close a resemblance as possible to the visual and/or auditory characteristics of an individual’s hallucinations (Craig et al., 2018, p. 33). Over a series of six weekly 50-min sessions, the patient engages in face-to-face work with the avatar, wherein the therapist facilitates a direct dialogue between the participant and avatar (Craig et al., 2018, p. 33). As the therapy progresses the avatar is engineered to gradually evolve from being persecutory to being supportive of the patient’s strengths (Craig et al., 2018, p. 33). All sessions are recorded on an MP3 player that the patient takes away to use at home, particularly in instances where the voices are heard (Craig et al., 2018, p. 33).

As Craig et al. (2018, p. 31) observe, voice-hearers typically find themselves in a submissive role in relation to their voices, a position that is characterised by feelings of inferiority and powerlessness which can reflect their experience of social relationships more generally. However, people who can establish a dialogue with their voices are often able to feel more power and control (Craig et al., 2018, pp. 31–32). A primary aim of avatar therapy is therefore to facilitate such a dialogue so that the voice-hearer can loosen the dominant (even omnipotent) grip of their voices (Craig et al., 2018, p. 31). In terms of the therapy’s efficacy, a recent single-blind randomised controlled trial found that the treatment led to “a rapid and sustained reduction in the severity of auditory verbal hallucinations by end of therapy at week 12 that was significantly superior to that achieved by supportive counselling” (Craig et al., 2018, p. 38). It was in fact observed that multiple participants, many of whom had failed to respond to extended courses of antipsychotic medication, “reported a complete absence of voices during the preceding week at the week 12 assessment,” with an even greater number experiencing such a cessation at 24 weeks (Craig et al., 2018, p. 37). Given that many of the participants in the study had been hearing voices for 20 years or more, such improvements should not be underestimated (Alderson-Day and Jones, 2018, p. 2).

Bringing these contemporary developments to bear on the Bionian theory explored, one could suggest that avatar therapy may function by allowing patients to ‘dream’ a persecutor that was previously only ever “coming out of the dark,” to quote Samuel Beckett, one of Bion’s own analysands (Oppenheim, 1994, p. 191). Furthermore (and in a manner close to how Pistiner De Cortiñas describes the clinical implications of ‘playing’), the process would also seem to facilitate the transformation of persecutory feelings experienced as “things in themselves” through the secure opening of a dialogue. While the underlying mechanisms remain largely open to debate, there are grounds to consider that this ‘playing’ may expedite a kind of ‘object creation’ that promotes the formation of meaningful relations to an internal world previously felt as both illusive and intrusive. In other words, through imaginative simulation that’s reinforced by the therapeutic apparatus, such hallucinatory presences may come to circumscribed and connected with, as opposed to being felt as uncontainably critical.

Thanks to the researchers conducting interviews with patients on the subject of their experiences while engaged in clinical trials of avatar therapy, there exists a substantial body of qualitative data from which to draw insights on the nature of voice hearing. One of the most significant findings insofar as this discussion is concerned is that, for many patients, the avatars and their voices come to represent feelings of low self-esteem that are related to past experiences of abuse and trauma (Craig et al., 2016, p. 49). Indeed for Romme et al. (2009, p. 25), in seventy per cent of individual cases the “voices are related to trauma and/or powerless making situations.” This finding is further supported by recent large-scale, general population studies that have indicated that the relationship between childhood trauma, psychosis and schizophrenia is “a causal one with a dose effect” (Read et al., 2005). As such, there could be said to be renewed empirical validation for Winnicott’s notion that psychotic symptoms should be regarded as a communication of the way in which the early environment failed. In less technical terms, for many of those experiencing the presence of voices that others don’t perceive, that very presence may be imbued with a childhood trauma and its perpetrators.

Evidence concerning how psychosocial factors such as child abuse and neglect can affect an individual’s likelihood of experiencing severe psychopathology raises further important considerations with respect to the nature and shape of the internal force that can, in such conditions, induce a profound fragmentation of the personality (Read et al., 2008, p. 235). When elaborating on the obliteration of psychic reality that’s synonymous with the psychoses, the Kleinian superego represents a crucial piece to factor into the puzzle. In Klein’s framework, the early superego is regarded as extremely severe, becoming less so in the process of development (Bott Spillius et al., 2011, p. 147). Crucially for this discussion, in pathological development, the severe early superego does not undergo modification; its pathogenic power may continue to be experienced in all its ruthlessness long beyond infancy. Bion would qualify Klein’s thinking yet further with his notion of a primitive psychic agency that asserts itself with the ruthless effect of being *“opposed to, and destructive of, all links”* (Bion, 1959, p. 314). In Bion’s understanding, this agency came to be explicitly defined as the “ego-destructive super-ego” and could be conceived of as responsible for attacking “links of emotion and reason between objects” (O’Shaughnessy, 2005).

Given the inherent difficulty in portraying the subjective experience of a fundamental disruption to meaning, locating material with which to apply this theory has a unique set of challenges associated with it. Nonetheless when consulting *The Centre Cannot Hold –* Elyn Saks’s autobiographical novel that documents a life suffering with schizophrenia – one is offered a rare and compelling glimpse of the internal dynamics involved in a formidable superego unleashing a dissolutive wave. Speaking from the perspective of her 8-year-old self, Saks writes:

*“My heart sinks at the tone of his [her father’s] voice: I’ve disappointed him. And then something odd happens: My awareness (of myself, of him, of the room, of the physical reality around and beyond us) instantly grows fuzzy… I think I’m dissolving… like a sandcastle with all the sand sliding away in the receding surf. This is scary, please let it be over! Most people know what it’s like to be seriously afraid… ‘disorganisation’ is a different matter altogether… One’s centre gives way.”* (Saks, 2007, pp. 12/13)

Having disappointed her father, an internalised version of whom comprises and configures her superego, Saks experiences the literal breaking down of physical and psychical reality. In theoretical terms, one might suggest that the passage illustrates the imposition and effect of a superego containing a “pure culture” (Freud, 1923, p. 53) of the death drive as described by André Green. More specifically, Saks’s superego here triggers the release of a visceral force that “delinks, fragments, and unbinds” meaning (Reed and Baudry, 2005, p. 132). Furthermore, as has been argued to be at the crux of psychotic functioning, there would in this instance appear to be the terrifying “disorganisation” of the very apparatus with which meaning is conferred.

As Beckett (1957, p. 93) writes in *Endgame* when reflecting on the multitude of moments that make up an existence: “Grain upon grain, one by one, and one day, suddenly, there’s a heap, a little heap, the impossible heap.” For Saks, it’s this “little heap” of a self that undergoes a collapse when the *“disobjectalizing function”* (Green, 2002, p. 646) of Green’s re-envisioned death drive pervades the psyche with entropic force. Unlike the connecting, investing and objectialising creative power of Green’s Eros that “links the infant with life, pleasure, the world of objects” (Reed, 2009, p. 5), in *The Centre Cannot Hold* we instead observe the “overtly psychotic” (Symington and Symington, 1996) disassembly of the psychic organs that ground the “centre” of the self. What’s more, given what’s already been explored in relation to the ways in which we come to bind psychic reality through the process of alpha-functioning, this disorganisation could be said to unravel and desolate the endopsychic contact-barrier between consciousness and the unconscious, thereby engendering “self-disappearance,” “disinvolvement” and severing all comprehension and link with reality (Green, 2002, p. 646).

## On the Contact-Barrier With Neuroscience

As has been observed by many of those with mutual allegiances to psychoanalysis and the neurosciences, the Bionian views hitherto explored can be seen to fit comfortably with “those emerging from the new neuroscience” (Lipgar and Pines, 2003, p. 194). Going beyond the mere alignment of theory, however, Bion’s insights into the nature of subjective experience arguably enhance the findings coming to light in this adjacent field (Lipgar and Pines, 2003, p. 194). Drawing on contemporary developments being pioneered at University College London – and chiefly utilising Karl Friston’s investigations into the Markov blanket – much of the remainder of this paper will examine how these interfacing disciplines co-inform each other’s understanding of the shaping and breaking of psychic reality. In advance of considering these synergies directly, an outline of the functional properties that Friston attributes to the Markov blanket is necessary in order to give body to the reflections.

For Friston (2013, p. 2), the Markov blanket has the fundamental property of inducing a “partition of states into internal and external states.” Widely applied in probabilistic machine learning, it is this conceptual structure that, in a statistical sense, allows for boundaried distinctions to be drawn between different systems (Kirchhoff et al., 2018, p. 1). Central to our concerns here is the fact that the Markov blanket formalises the separation “between an inner and an outer environment” (Pezzulo and Levin, 2018, p. 32). In terms of a basic illustration, the cell represents an intuitive example of a living system with a Markov blanket; unless in possession of such a boundary, the cell would cease to exist as there would be no way of distinguishing it from the environment in which it lives (Kirchhoff et al., 2018, p. 2). Following this reasoning to its logical conclusion, evidence for any biological system is thus said to be contingent on it having a Markov blanket that facilitates the definition of inner from outer and which therein allows for the organism to be differentiated from that which it is not (Kirchhoff et al., 2018, p. 2). At the human level with which this paper is concerned, the Markov blanket is thus broadly conceived of as forming a sensory boundary, the activity beyond which consciousness has not been extended to.

Adding a further level of detail, internal states – the activity of which establishes the Markov blanket’s existence – can themselves be subdivided into *sensory* and *active* states (Kirchhoff et al., 2018, p. 1). Sensory states are, in Friston’s terms, defined as those that are caused by external states and which influence, but are not influenced by, internal states (Kirchhoff et al., 2018, p. 3). An example of this would be sensory information which is mediated by sensory states as it gets from the outside world, into the internal world (Friston, 2017). Active states, on the other hand, proceed in the opposite direction; they are caused by internal states and they influence, but are not themselves influenced by, external states (Kirchhoff et al., 2018, p. 3). An important consequence of this understanding is that the outer environment can only be seen “vicariously by the internal states, through the Markov blanket” (Friston, 2013, p. 2). It is for this reason that Friston refers to the Markov blanket as constituting a “veil” through which we infer the external causes of our sensory impressions (Friston, 2014a). Moreover, in addition to functioning as a metaphorical veil that discerns the sense impressions landing upon it, the Markov blanket also operates as a “projection screen” onto which are cast the habitual mechanisms (mediated by active states) that we use to *make sense of* the world (Friston, 2014a).

According to the exponents of free energy neuroscience, “active Bayesian inference” represents the chief mechanism by which we discern the sense impressions that come into contact with the Markov blanket (Friston, 2013, p. 1). Used for “calculating conditional probabilities,” Bayesian inference involves creating and testing hypotheses, and updating beliefs in accordance with whether or not these predictions correspond to the data sampled (Joyce, 2008). Put simply, in instances of “prediction *error*,” what is expected to occur is invariably at odds with what is actually experienced. In light of such observations, a Bayesian system would necessarily revise itself in order to make more accurate predictions in the future. Cast in the terms already defined, this process corresponds to an organism attempting to preserve its existence by developing, maintaining and updating a “generative model” of its external environment (Kirchhoff et al., 2018, p. 5). Moreover, it is from within the periphery of the Markov blanket that the brain, functioning as a Bayesian machine, continually monitors the extent to which its internally constructed models accurately reflect the external reality that it stands in causal relation to. One of the most profound and over-arching implications of this with respect to human beings is that it revalidates Kant’s notion that ‘*our manifest conscious image of ourselves as self-aware subjects of experience… is internal to our minds’* (Hopkins, 2012, p. 236).

Grounding all of this in psychoanalytic thinking, the way in which we come to know whether our generative model (and its constituent set of predictions about external states) is accurate or not is through affective feeling (Solms, 2014). Given that our perception of reality is all in the service of meeting our needs in that reality, (which Freud wrote about in terms of it resulting in “an *experience of satisfaction*”), possessing an inaccurate generative model of the world would mean that needs remain unmet and affects come into play as a way of enforcing a revision to the model (Solms, 2014). When Friston therefore speaks about “minimising prediction error” and giving up on predictive models that don’t correspond to external states, he’s referring to Freud’s reality principle, albeit in a different frame of reference (Solms, 2014). It is precisely this minimisation of prediction error – which results in a diminution of distressing affect – that ultimately sustains survival.

Adding another layer of detail so as to be able to comprehensively apply Bion’s ideas, reducing prediction error (and therein conforming to the reality principle) are said to equate directly to the minimisation of “free energy” (Friston, 2014b). For Friston, minimising free energy is a defining trait of any biological system capable of preserving its existence over time (Friston, 2013, p. 2). Crucially and in the context of the preceding discussion, free energy is precisely the same quantity that is optimised (toward a minimum level) in Bayesian inference (Friston, 2013, p. 1). As such, an abundance of free energy – also known as “surprise” – would, in human beings, signal an individual making inaccurate predictions in relation to the world around them (Friston, 2014b). In psychoanalytic language, this translates as deficient reality-testing. In contrast, the process of resolving prediction errors and instigating effective reality-testing is, within Friston’s paradigm, conceived of as involving the conversion of free energy into “bound energy” (Friston, 2014b). It is this “binding” that occurs within the boundary established by the Markov blanket and is described as fundamentally requiring the existence of “higher structures” in the organism (Friston, 2014b).

A principal consequence of this binding is that it allows the organism to operate in opposition to that which is “the long-term average of surprise”: entropy (Friston, 2013, p. 2). By placing an “upper bound” on the entropy or dispersion of sensory states – (while simultaneously using those sensations to infer the external states of the world) – the organism in possession of a Markov blanket is thus able to “resist the second law of thermodynamics” (Friston, 2013, p. 2). Defined as the way in which isolated systems always evolve toward a state of maximum entropy, a resistance to this law within biological systems has the fundamental effect of allowing them to “preserve their functional and structural integrity” (Friston, 2013, p. 1). For systems that are incapable of resisting dispersion (and which therefore do not minimise free energy), the entropy of their sensory states “would increase indefinitely – by the fluctuation theorem,” ultimately meaning that they “cannot exist” (Friston, 2013, p. 2). Moreover, not only does this vital ability to operate in opposition to entropy enable the continued existence of living systems, certain corollaries of it also facilitate their flourishing; as Friston (2013, p. 1) describes, evading dispersion allows for “homeostasis and a simple form of autopoiesis,” the latter of which – as was alluded to earlier in this paper in relation to alpha-functioning – refers to a system capable of reproducing and maintaining itself. Appropriating the words of Zizek (2012, p. 467), we might therefore regard the Markov blanket as expediting “the gradual rise of order out of chaos.”

## Superimposing the Conceptual Frameworks

As was stated at the start of the paper, the concepts explored will now be examined from the perspective of where they intersect one another. Given the limitations of this paper however, compared to the scope of the material under discussion, one can only hope to present more questions than answers. As such, a series three suggestions will be posed with accompanying discussions, each of which will incorporate elements of understanding from the psychoanalytic and neuroscientific theories considered.

### Is There an Intimate Connection Between the Interoceptive Contact-Barrier and the Exteroceptive Markov Blanket?

The suggestion here is that Bion’s (1962a, p. 16) caesura which produces “ordered thought” by marking “the point of contact and separation between conscious and unconscious elements” (Bion, 1962a, p. 17), may potentially have a parallel correspondence with the Markov blanket that establishes “generalised synchrony” through using internal states to encode “events in the external world” (Friston, 2014a). Isolating the dynamics of the connexion more specifically, the infant’s endopsychic contact-barrier which is constructed to facilitate the binding of beta-elements, could be conceived of as later projected out onto external reality, much in the way that Freud (1923, p. 26, my emphasis) describes the ego as the “*projection of a surface.*” Drawing on the ideas of Didier Anzieu, the suggested organisation might thus be said to resemble a “psychic envelope” (Jacobus, 2005, p. 9). In this frame, the contact-barrier that compounds interoceptive chaos by virtue of dream-work-alpha is projected out on external reality, forming a “visual dream-film” (Jacobus, 2005, p. 9) that functions mimetically to bind exteroceptive input. Through this view, we learn to work with the world having learnt to work with ourselves.

Such a conception of the “psychic envelope” is highly compatible with seeing the mind itself as a *container* (Hopkins, 2000, p. 8). Indeed, the English language has an abundance of analogies that instinctively pertain to precisely this understanding. From a forgetful person being described as having a “brain like a sieve,” to an unstable individual being thought of as “out of their mind” or “having gone to pieces,” there exists an entire family of metaphors that refer to a notion of the mind being circumscribed by containing boundaries (Hopkins, 2000, pp. 8–9). As has been demonstrated in relation to Bion’s writing moreover, such individual psychic containment is made fundamentally possible by the dualism of a container-contained relation. While, we may therefore learn to work with the world having learnt to work with ourselves, as Vygotsky (1998, p. 170) observes, we actually *become ourselves through others*.

In terms of visualising the structural organisation of these interoceptive and exteroceptive boundaries, the infamous analogy of Plato’s cave involves an imagined space and components that are particularly compatible with the proposed understanding (Solms, 2014). Without wishing to map the analogy on in too concrete a manner, its philosophical implications could yet be seen to speak directly to some of Friston’s basic proposals. In Plato’s “strange image,” multiple prisoners sit facing the wall of a cave, unable to move (Plato, 360 B.C.E). Behind them is situated a fire, in front of which is a walkway where unseen men patrol carrying statues (Plato, 360 B.C.E). For these hypothetical prisoners, all that’s ever perceived are their own shadows and those cast by the statues “which the fire throws on the opposite wall of the cave” (Plato, 360 B.C.E). Despite the analogy progressing, it is this crucible that’s of interest in this instance.

Of particular note is the extent to which the prisoners in Plato’s theoretical cave are illustrative of the fact that, as Friston states, “we are only seeing our projections” (Solms, 2014). In fact, at the 2014 Sandler Conference, Friston went as far as to specifically describe the Markov blanket as a “projection screen” (Friston, 2014a). Quoting the 19th century physicist, philosopher and physician, Hermann von Helmholtz, he unambiguously stated that objects “are *imagined* in the field of vision to account for sensation” (Solms, 2014). Importantly for our concerns, Friston’s model here echoes something fundamental to a psychoanalytic understanding of how individuals come to perceive the world around them: “they imagine a construction of the world,” and yet this fantasy must account for actual sensation (Solms, 2014). Bringing in Bion, one might also add that these imagined objects are irrevocably coloured by phantasmagorias of the imagos that conjugate at level of the contact-barrier. In terms of a Platonic parallel, the statues that cast the shadows would be analogous to these imagos.

### Might a Disobjectalising of the Contact-Barrier Be Reflected as a Tear in the Functional Fabric of the Markov Blanket?

As is evident from the significance of the processes associated with the Markov blanket, the structure carries a particular importance for the psychoanalytic understanding of how a person might experience a “loss of contact with reality,” as Freud put it (Freud, 1924, p. 183). While Freud’s terse description of psychosis remains an important gateway into considering the condition, bringing Bion together with Friston adds new dimensions to the understanding of the processes involved in such a ‘loss of contact.’ As Bion (1962a, p. 16) writes, “alpha-function, which makes dream possible… preserves the personality from what is virtually a psychotic state.” Given the suggested interrelation between the structural layers of the “psychic envelope,” it would therefore make sense to contend that damage to that which is the product of alpha-function – the contact-barrier – would correspondingly affect the operation of the projected surface: the Markov blanket.

As was explored in the context of the ego-destructive superego, damage to the contact-barrier may be the result of a Greenian death drive critically interrupting “relationships in the activity of the mind” (Green, 2010, p. 29). Furthermore, in circumstances where this psychic force “dissolves connections” (Reed, 2009, p. 5) at the level of the contact-barrier, then – *due to the projection of the surface* – we’d see the Markov blanket’s composition and concordance necessarily implicated. As such, we can begin to perceive a direct relationship between a contact-barrier that is disobjectalised by a suffusion of the drive toward “neuronal inertia” (Freud, 1895, p. 296), and a Markov blanket that is ‘torn’ and thus ineffective.

In Friston’s terms, a radical lack of the functionality that’s otherwise induced by a structurally integral Markov blanket would result in a proliferation of unbound free energy. As discussed, this equates to massive prediction errors being made in relation to how the external world is expected to behave. Moreover, as Solms (2014) points out in no uncertain terms, “minimising prediction error *is* the reality principle.” Therefore for the individual with an impaired capacity to minimise prediction error, “banging into” the aspects of reality that couldn’t or wouldn’t be sampled by the dysfunctional apparatus is likely to be a recurrent phenomenon (Solms, 2014). As Freud (1924, p. 185) puts the observation in his paper on *The Loss of Reality in Neurosis and Psychosis*, “in a psychosis the rejected piece of reality constantly forces itself upon the mind.” As such, the psychotic experiences the return of the disavowed as the rejected pieces of reality repeatedly puncture the individual’s distorted worldview (Quinodoz, 2005, p. 245). Of course, given what’s been discussed, one could argue that our perception is always already distorted to some degree. Nonetheless, the rupturing of the contact-barrier through the “work of the negative” (Green, 1992, p. 586) would undoubtedly represent psychopathology of another calibre.

In extremely severe circumstances, an overwhelming profusion of free energy resulting from such a rupture would mean that the entropy of the individual’s “sensory states would not be bounded” (Friston, 2013, p. 2). In other words, and by way of a more tangible example of how this might be experienced, Elyn Saks’s aforementioned depiction of *“dissolving… like a sandcastle with all the sand sliding away”* gives a visceral impression of sensory states quite literally dissipating. Having been explored already in relation to Green’s death drive, Saks’s passage also speaks directly to Friston’s assertion that an individual experiencing the wholesale disintegration of their Markov blanket would consequently fail in their attempts to minimise dispersion (Friston, 2014a). In such a scenario, an individual’s “autopoietic maintenance” is crucially said to be at stake (Friston, 2014a). This process was similarly identified and explained in the first section of this paper as a key feature of alpha-functioning. Consequently, while correlation doesn’t automatically mean causation, the numerous similarities between these interior and exterior surfaces would seem repeatedly to point to the existence of a reciprocal relationship between them.

### What Are the Clinical Implications of Working at the Level of the Projected Surface?

Working at this level could be seen to be precisely what’s afforded by the psychoanalytic technique of probing the patient’s transference. Utilised extensively in the clinical setting, the phenomenon is said to offer psychoanalysis “the inestimable service of making the patient’s hidden and forgotten erotic impulses immediate and manifest” (Freud, 1912, p. 107). As Sandler (1976, p. 43) writes, the transference can therefore be regarded as comprising “a concealed repetition of earlier experiences and relationships” which are thus revived and projected onto the analyst. In terms of the question of which experiences and relationships we might expect to see revived and projected, Freud (1936, p. 18) identifies them as having “their source in early – indeed, the very earliest – object relations.” Provided the analyst can contain the patient’s projections through well-timed and accurate interpretations, however, there exists the possibility of these unconscious ways of relating being transformed into self-knowledge. In the words of Ferenczi (1933, p. 160), the patient that’s contained in this way stands to “re-experience the past no longer as hallucinatory reproduction but as an objective memory.”

Returning once more to avatar therapy, this pioneering method could be argued to represent a profound intensification of the transferential process that’s facilitated by the classical analytic setting. By going beyond the transferral of internal objects, to the point of creating and projecting them into an avatar, this method of treatment facilitates a process whereby these hallucinatory and delusory presences are engaged in the form of externalised and newly recognisable imagos. Moreover, by contrast to the internal persecutory presence, an external avatar represents a persecutor securely contained and controlled. As was explored earlier in this paper, while these persecutors may be imbued with a childhood trauma and its perpetrators, in this specialised clinical scenario, they’re disarmed of their ability to cause unrestricted damage. As such, Ferenczi’s (1933, p. 160) psychoanalytic proposal that there must be a vital “*contrast between the present and the unbearable traumatogenic past*” would appear to have been here maintained.

Building on the suggested model of interrelated psychic surfaces, these moderated and virtually represented avatars – once introjected – could be said to function as clusters of contained affect around which new conjugations of alpha-elements may cohere. Viewed through the prism and language of Bion, the technique might thus be argued to allow for the formulation of more resilient coping strategies by enabling internal persecutors to be rendered increasingly accessible on the intra-psychic level of ‘dreaming.’ It’s in this sense that capturing and engaging internal phenomena at the projected surface (in order to explore them securely and therapeutically), may have the corresponding effect of enhancing the person’s interoceptive “sense organ for the apprehension of psychical qualities” (Freud, 1900, p. 574).

## Conclusion

From Freud and Klein, to Green and Winnicott, Wilfred Bion’s writings intersect and inform countless of the theories developed by his peers. Indeed, the contributions that Bion brought both to his own and other disciplines are far from static; they continue to unfold in new ways in accordance with emerging concepts and evolving modes of thought. As has been explored in this paper, the interface that exists between *Learning from Experience* and contemporary Fristonian neuroscience is the location of a particularly fruitful cross-fertilisation of ideas. While demonstrating the points where these rich veins of thought make contact has been the ultimate goal of this paper, due to their depth and complexity, there’s undoubtedly more work to be done. Rather than drawing premature conclusions therefore, it is hoped that various openings have been indicated.

Reflecting more specifically, the esoteric ways in which mental life is shaped were considered as conspicuously revealed by an understanding of how psychic elements and functions move between container and contained. For Bion, it’s as a result of this process that we form a receptive internal world in which conscious and unconscious elements are both separate and in communication. At the very crux of this organisation lies the ability to ‘dream,’ the implications of which centrally include the digestion of emotional experience and a propensity for regenerative growth. By contrast, failures of this capacity and the attempt to avoid the potentially painful perspectives it induces have been investigated in relation to severe psychopathology.

The continued relevance and flexibility of these theories testifies to the true extent of Bion’s inter-disciplinary potential; nowhere is this clearer than when his concepts are brought into dialogue with Friston’s proposals. As a result of this synthesis and through the applied use of the relationship between interoceptive and exteroceptive contact-barriers, profound therapeutic gains stand to be made. In this regard – and with technological advances making object creation at the level of the projected surface increasingly feasible – it’s possible to conceive of further developments in how disturbed internal boundaries may be approached and reconstructed. As this work has endeavoured to show, it is through fostering these frontiers of consciousness that the dream of a contained unit self is made manifest.

## Author Contributions

MM is responsible for the work associated with the production of this paper.

## Conflict of Interest Statement

The author declares that the research was conducted in the absence of any commercial or financial relationships that could be construed as a potential conflict of interest.

## Footnotes

1 *Caesura*: Bion extended the term that he borrowed from Freud to a complex notion of gap, fissure, space, and bridge, having the function of both separating and communicating (Pistiner De Cortiñas, 2011, p. 129).

2 *Autopoiesis*: An autopoietic organism or machine is defined as one that “continuously regenerates and realizes the network of processes that produced them” (Maturana, 1973, p. 78).
